# Mobile phone infrastructure provides evidence of improved HIV viral load monitoring in Malawi

**DOI:** 10.1371/journal.pdig.0001094

**Published:** 2026-01-21

**Authors:** Rachel Haggard, Christopher Mwase, Brandon Klyn, Lynn Metz, Tyler Smith, Hannah Cooper, Brown Chiwandira, Dylan Green, Linley Chewere

**Affiliations:** 1 Cooper/Smith, Austin, Texas, United States of America; 2 Cooper/Smith, Lilongwe, Malawi; 3 Department of HIV, STIs and Hepatitis, Ministry of Health, Lilongwe, Malawi; Karolinska Institutet, SWEDEN

## Abstract

Malawi has 991,600 people living with HIV and has expanded access to annual HIV viral load testing to enhance care quality for clients. However, significant delays persist in returning viral load (VL) results back to facilities and to clients. To address this, we implemented a digital VL results return (VLRR) application, using existing mobile phone platforms to expedite results return to clients and healthcare providers (HCPs).VLRR is a digital SMS/USSD platform leveraging mobile phones to reduce turnaround time (TAT) and improve access to VL results. To evaluate the VLRR intervention, we: (1) estimated the TAT for digital results return, (2) calculated open rates of digital results, (3) conducted a mixed methods evaluation with VLRR users, and (4) estimated the potential cost savings from avoiding unnecessary sample redraws. From April 2022 to June 2024, HCPs registered 4,067 clients. For each client, TAT was calculated separately for the periods before and after enrollment in the VLRR system. On average during this period, clients received results in 128 days before VLRR enrollment and 48.5 days after enrollment, reflecting a 62.4% improvement. By July 2023, VLRR clients and HCPs received results in an average of 30 and 38 days. The overall open rate for digital results (opened by either a client or HCP) was 60% and nearly 100% of clients and HCPs indicated they wanted to the application to continue. Lastly, if VLRR were scaled nationally, it has the potential cost savings of $1.8-6.7 million USD.VLRR is effective in reducing TAT and improving access to VL results. To enhance uptake and achieve national scale, VLRR can be integrated into Malawi’s existing EMR systems, further reducing TAT and enabling HCPs to deliver higher quality care and improve clinical outcomes.

## Author summary

The digital VLRR application uses SMS and USSD on a mobile phone to deliver viral load test results back to clients and healthcare providers for those clients who opt into receive their results digitally. A major challenge for HIV positive clients in low-income settings is monitoring their care properly due to long turnaround times from blood sample to receiving their paper test results back at a facility. The option to receive results digitally reduces the turnaround time of results by more than 60% because it cuts out the time it takes to deliver paper results from lab to clinic and clinic to client. This has both positive clinical and monetary implications for clients because they can have more agency to monitor their care in a timely manner.

## Introduction

Timely turnaround times (TAT) for diagnostic results from laboratories to healthcare providers (HCPs) and clients are essential for quality care [1–3]. Delays and backlogs in laboratory results hinder timely diagnosis and treatment [4]. Excessive backlogs can render results clinically irrelevant, requiring blood sample redraws and restarting the process. This increases laboratory workload and strains the healthcare system both in time and financial resources [5]. Faster TAT is crucial for effective client management [4].

HIV is the leading cause of death and disability in Malawi, with approximately 991,600 people living with HIV (PLHIV), representing 4.6% of the population [6]. Viral load (VL) testing has been the gold standard for monitoring antiretroviral therapy (ART) adherence since 2013, when the World Health Organization (WHO) released updated ART guidelines [7]. Malawi’s standard of care recommends a VL test six months after ART initiation and, if virally suppressed, every year thereafter, with results delivered to facilities within 21 days and to clients within 30 days [8]. However, Malawi has only 14 specialized laboratories to process VL samples for clients across 850 + ART facilities, leading to long processing times, laboratory backlogs, and extended travel times to clinics [9]. As a result, these standards are rarely met, impacting the quality of care for PLHIV and long-term client outcomes. To meet the Joint Programme on HIV/AIDS (UNAIDS) treatment target of greater than 95% viral suppression among PLHIV on ART [10], Malawi must innovate its laboratory information systems and significantly reduce TAT for VL testing.

There is evidence that providing laboratory results directly to clients via mobile phone technologies can reduce negative clinical interactions and mitigate related consequences [11–14]. Further to that, Malawi sought to amplify the undetectable = untransmittable campaign by implementing a VL results return system that directly notifies clients of their undetectable status, empowering them with knowledge and promoting HIV prevention. This approach also empowers clients to seek the services they need to manage their condition. Zambia and Zimbabwe have piloted short message service (SMS) interventions that sent results to facilities and reminders to clients to visit the facility for their results [11–13]. In South Africa, the National Health Laboratory Services piloted a mobile platform called iThemba, a smartphone application that delivered HIV VL results, education, and clinical support directly to users’ smartphones [14].

These interventions demonstrated that the use of mobile phone technologies to deliver results to clients can improve the client experience [11–14]. Providing clients with their results via mobile phone can limit the potential for negative encounters, provide positive encouragement, reduce delay in knowledge about one’s health status, and increase health-seeking behavior, which has a direct effect on longevity and the quality of a client’s life [15]. We set out to determine the feasibility and acceptability of sending VL results to healthcare providers and clients using a combination of SMS and unstructured supplementary service data(USSD). SMS is what allows individuals to receive text messages on their phones and USSD allows users to access services on their phones without requiring internet connectivity [16]. USSD allows users to interact with their phone and choose different options from a variety of menus. For example, with mobile banking USSD, a user can get their bank account balance or send money to another person via a two-way communication of information [16]. To our knowledge, no country has leveraged both SMS and USSD-based technology to transmit results. Subsequently, most countries did not report an open rate of the SMS or application. Similarly, no applications targeted the system-wide benefit that a mobile phone results return platform could have on the overall efficiency of the laboratory system.

We hypothesized that a cost-effective SMS/USSD platform that relayed confidential and timely results return could be created for all phone users, both feature (basic phones that have 3 communication channels only (e.g., use calls, SMS, and USSD) and smartphone, in Malawi to reduce TAT. To test this hypothesis, we developed a digital SMS and USSD-based platform to deliver VL results directly to clients and HCPs to reduce TAT and improve access to VL results. We evaluated this in four ways: 1) by estimating the TAT, 2) by estimating the result open rate, 3) through a formal mixed methods evaluation of the clients and HCPs using the application, and 4) by evaluating the system-wide benefit of the application by estimating the number of redraws that could be offset through near-real-time results return.

## Methodology

The VL Results Return (VLRR) application was launched as a pilot in February 2022 in four health facilities across Malawi. VLRR was extended to 5 additional sites in October 2023 and an additional 5 sites in January 2024. We used a mixed methods approach to evaluate the platform’s success at all 14 health facilities across 13 districts.

Guided by the Ministry of Health, we selected study sites based on the 3 regions of Malawi, rural/urban status, ART cohort size (>900 clients), affiliation (public vs private/semi-private), and access to electronic medical records. We enrolled clients who were above 18 years old, due for a routine VL test, and they consented to receive their VL result through a mobile phone at the selected study sites.

We used quantitative methods to determine the feasibility of the intervention to effectively return results by estimating the TAT for both clients and HCPs. We conducted a comparative analysis between the proportion of clients accessing results using VLRR compared to the paper-based system (e.g., result open rates). We used mixed methods to determine the acceptability of the platform through structured surveys with both clients and healthcare providers. The in-depth interviews with healthcare providers and clients gauged their perception of the platform’s ease of use, accessibility, user support, privacy, and security.

Lastly, we conducted a cost analysis of the sample redraws required due to long TAT. How we define and estimate the VLRR outcomes can be found in Table 1.

**Table 1:** VLRR outcomes.

Outcome	Definition	Estimation
VLRR TAT	The number of days it takes from sample collection to when a result is opened digitally by a client or HCP.	Summation of total # of days from sample collection to result opened
Open Rate	The percentage of tests that had an SMS notification that a result was ready and were opened digitally by a client or HCP.	(# test results with an SMS notification sent and opened digitally by a client or HCP)/ (# of tests results with an SMS notification sent) * 100
VLRR Acceptability	A mixed methods assessment on user perception and feedback of the platform.	In-depth interviews
VL Test Redraws	The total number of samples taken in a year that were redrawn due to clinical irrelevance. Clinically irrelevant VL result is defined as a result delivered to the facility after 3 months from sample collection since it can no longer be used to inform the care of the client. Note: the numerator has 5 months rather than 3 months to provide a conservative estimate acknowledging that in some cases, clients return late for their VL samples or results older than 3 months may be utilized by an HCP, despite what is recommended.	(# of clients with low VL and two tests within 5 months for the 11 EMR enabled sites)/ (total # of clients with low VL) * 100
Cost savings due to redraws	The amount of money that could be saved through VLRR by estimating the number of unnecessary VL test redraws caused by clients not receiving their results within three months.	Lower Bound: (total # VL tests per year) x (% of tests redrawn due to processing delays) x (minimum commodity cost of VL test) Upper Bound: (total # VL tests per year) x (% of tests redrawn due to processing delays) x (comprehensive cost of VL test)

### SMS/USSD application intervention

**Fig 1:**
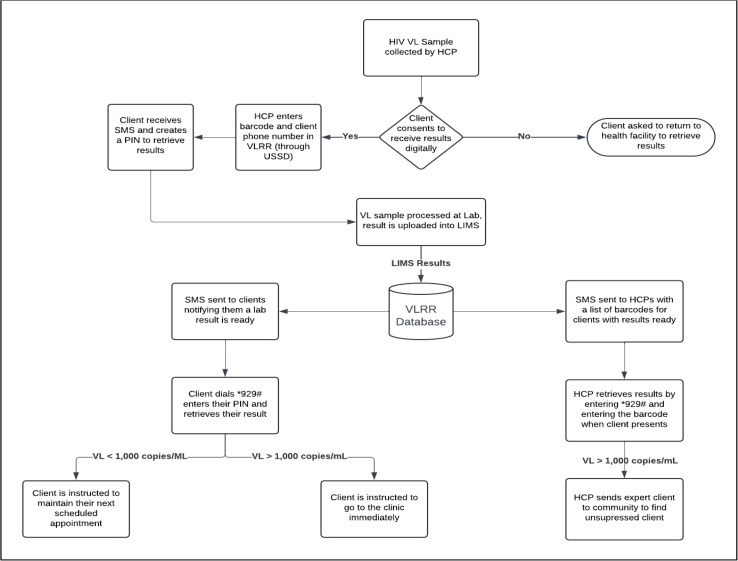
VLRR workflow for clients and HCPs.

The VLRR application is a digital SMS and USSD-based platform that leverages mobile phones (both feature and smartphone) to deliver results directly to clients. HCPs at facilities who provided HIV services were also notified via SMS that clients’ results were ready, and they could also view the results by logging into the USSD platform. This was intended to reduce the TAT and improve access to VL results by circumnavigating some of the challenges within the paper-based system (e.g., lost results, lab backlog reduction, result delivery times, client travel time). We leveraged a combined SMS and USSD platform to make it accessible for non-smartphone users. Clients without a phone were still eligible to participate, in these cases results would only be provided to HCPs. We used SMS to send the notifications that a result is ready and USSD to privately check results. Results were only viewed within USSD to protect client privacy and confidentiality, as USSD times out after 30 seconds if not used. We used routine Laboratory Information Management System (LIMS) and VLRR data for these analyses. The LIMS database houses routine VL results from all 14 laboratories in Malawi. We connected VLRR platform’s Application Programming Interface to the LIMS database. When a laboratory approves a result in LIMS, it is synced to VLRR which triggers a nondescript SMS to be sent directly to a client’s phone notifying them their result is ready. The SMS prompts the client to dial the *929# shortcode to check their VL result in the USSD platform. Importantly, when clients accessed their results through USSD, the platform provided not only the result but also a clear, easy-to-understand interpretation and a directive on the appropriate next steps. The *929# shortcode was granted to the Ministry of Health by the Malawi Communications Regulatory Authority. It was initially utilized for COVID-19 and was extended to other digital services, including VLRR. Additionally, the Ministry of Health supported the process of connecting VLRR to the two most used telecommunication companies in Malawi. Fig 1 shows the VLRR workflow for clients. Additionally, HCPs who were trained on and use the VLRR platform receive a daily SMS, Monday through Friday, with a list of samples that have results ready. An HCP checks a client’s result within the USSD platform using the client’s ART number. If the VL result is high, the HCP is prompted to send out a community health worker or an expert client to retrieve the client in the community and bring them back to the clinic.

#### 1) Turnaround time.

When a client on ART gets a VL sample taken at the facility, the HCP asks them if they want to receive their results digitally. If the client consents, the HCP enrolls them into the VLRR platform, helps the client set up their personal identification number (PIN) in the USSD platform, and teaches the client how to navigate the USSD platform. The client learns how to dial the USSD shortcode, enter their PIN, and check their VL results through this process.

We used TAT and open rates to determine the successful delivery of results by the platform to clients and HCPs. We analyzed routine data collected by the platform throughout the pilot period. We calculated the TAT from sample draw to when a result was opened by a client and a HCP. We monitored and calculated TATs throughout the lifecycle of the intervention and compared the percent change between the status-quo TAT (pre-VLRR) and VLRR’s TAT. To estimate the status quo national TAT, we used electronic medical record (EMR) data from 249 EMR sites from January 2019 to May 2024. We also subset EMR data to the 11 VLRR EMR sites to estimate the status quo TAT for VLRR sites only. Three VLRR sites were non-EMR-enabled sites, so we could not calculate a status quo TAT for these sites. VLRR TAT’s were still calculated for these non-EMR-enabled sites, however they were excluded from the pre-and post-VLRR enrollment TAT analysis, as no pre-enrollment data were available.

#### 2) Application open rates.

To determine open rates, we tracked the number of clients and HCPs who were sent an SMS notifying them that a result was ready. Open rates for VLRR were calculated as the percentage of clients or HCPs who viewed the result on USSD out of the total number of samples where an SMS notification was sent that a test result was ready. We compared the open rates for VLRR with those of existing results return digital platforms in sub-Saharan Africa and high-income countries to determine the performance of our application.

#### 3) Mixed methods evaluation of the application.

We engaged four Malawian research assistants (RAs) to conduct structured and semi-structured interviews with clients and HCPs. These research assistants had previous experience with mixed methods research and public health and attended an in-depth training on the survey tools.

They interviewed HCPs who were trained on and used the VLRR platform. We aimed to interview 5 HCPs per site. However, some sites contributed lower numbers as some providers were away on leave or assigned other tasks, thereby limiting their availability for interviews. We interviewed a total of 67 healthcare providers. Table 3 shows the descriptive statistics for HCPs included in the mixed-methods study including sex breakdown, average age, average time working in HIV, and average period using the platform.

**Table 3:** Site level VLRR characteristics, open rates, and TATs.

Pilot phase	Rural/Urban	District	Site ID	Samples registered	Results sent	Results opened by clients	Results opened by HCPs	VLRR TAT for Q2 2024 - days (opened by HCP or client)	EMR enabled
Initial	Rural	Lilongwe	Site 1	528	479 (91%)	78 (16%)	269 (56%)	40	Yes
Initial	Rural	Mzimba	Site 2	117	111 (95%)	14 (13%)	89 (80%)	25	Yes
Initial	Rural	Machinga	Site 3	205	189 (92%)	26 (14%)	94 (50%)	34	Yes
Initial	Urban	Lilongwe	Site 4	788	689 (87%)	175 (25%)	242 (35%)	45	Yes
Scale-Up	Rural	Mulanje	Site 5	419	328 (78%)	10 (3%)	104 (32%)	71*	Yes
Scale-Up	Rural	Kasungu	Site 6	112	78 (70%)	23 (29%)	59 (76%)	35	No
Scale-Up	Rural	Neno	Site 7	44	37 (84%)	6 (16%)	30 (81%)	42	No
Scale-Up	Rural	Salima	Site 8	70	62 (89%)	12 (19%)	53 (85%)	44	Yes
Scale-Up	Rural	Dedza	Site 9	259	230 (89%)	20 (9%)	47 (20%)	32	No
Scale-Up	Urban	Zomba	Site 10	489	464 (95%)	228 (49%)	195 (42%)	23	Yes
Scale-Up	Urban	Rumphi	Site 11	161	149 (93%)	35 (23%)	55 (37%)	41	Yes
Scale-Up	Urban	Chiradzulu	Site 12	226	207 (92%)	73 (35%)	149 (72%)	25	Yes
Scale-Up	Urban	Mchinji	Site 13	457	380 (83%)	124 (33%)	353 (93%)	63	Yes
Scale-Up	Urban	NkhataBay	Site 14	192	181 (94%)	65 (36%)	46 (25%)	36	Yes
	Total			4067	3584 (88%)	889 (25%)	1785 (50%)		

*Data from Q1 2024, as no samples were registered in Q2 2024 in Mulanje.

We aimed to interview 10 clients per health facility. However, some facilities contributed fewer numbers of clients. Reasons for the fewer numbers included a lack of interest to participate in the interviews, transportation challenges in certain areas due to flooded rivers, and prioritization of farming activities over coming to the facility for interviews. We obtained clients via convenience sampling. HCPs reached out to clients who were enrolled in the VLRR platform and asked them of their interest to participate in the interviews. We interviewed a total of 117 clients. Table 4 shows the descriptive statistics of the clients interviewed across the 14 sites.

**Table 4:** Descriptive statistics of HCPs (N = 67) and clients (N = 117) using VLRR.

Variable	HCP		Client	
Females Interviewed	32	48%	67	57%
Males Interviewed	35	52%	50	43%
Median Age [range]	39	23 – 59	44	18 – 74
Median Years Working in HIV [range]	9	0.25 – 20	N/A	
Median Years on ART [range]	N/A		12	0.5 – 26
Median months using VLRR [range]	4	1 – 24	4	0.25 – 24

Interviews were conducted in a private room at the health facility and only included the participant and the RA. Before each interview, the RAs obtained written consent from all study participants. If a participant could not read or write, the RAs read out the consent form to the participant and obtained written consent using a thumbprint. The RAs conducted the interviews using questionnaires and voice recorders. The questionnaires for the clients and HCPs were different and consisted of a combination of qualitative and Likert scale questions. All questionnaires were verbally administered to healthcare providers in English, while clients were provided the option of being interviewed in either English or Chichewa. All interviews conducted in Chichewa were recorded, translated, and transcribed into English.

For clients, the survey evaluated clients’ favorability of the platform and whether they would recommend VLRR to others living with HIV, ease of accessing the results and overall perceptions of VLRR, and any privacy and security concerns. For HCPs, we evaluated how they felt VLRR supported clients, the challenges they faced with the paper-based process and whether VLRR addressed those challenges, ease of use of the platform, VLRR’s effect on their overall workload, and continuity of VLRR.

We used two coders to manually code all qualitative questions in Excel. The coders used inductive coding through thematic analysis to build a codebook. Once the codebook was developed, we analyzed the coded responses to identify recurring patterns develop overarching themes. All responses were categorized within a theme. These categories were not mutually exclusive; therefore, a single respondent could have an answer that fits into several categories.

#### 4) Cost analysis - redraws.

To quantify the potential cost savings from unnecessary sample redraws, we used LIMS data to calculate the total number of samples taken in a year that were redrawn due to clinical irrelevance. Clinically irrelevant VL result is defined as a result delivered to the facility after 3 months from sample collection since it can no longer be used to inform the care of the client. It is necessary to note that current data systems in Malawi, including LIMS and the EMR, only track redraws due to laboratory error. These systems do not capture redraws necessitated due to clinical irrelevance.

To estimate the number of tests that required a redraw due to clinical irrelevance, we calculated the proportion of clients with a low VL who had two tests within 5 months using EMR data for the 11 EMR-enabled sites. We chose this period because the Malawi Integrated Guidelines for Clinical Management of HIV 2022 [17] indicate that a client with a low VL should receive a VL test no earlier than 6 months after their last low VL test.

We then estimated the all-in cost per VL laboratory test from the lowest to the highest possible costs. Based on an assessment conducted by the Médecins Sans Frontières [18], the all-in (e.g., test, labor, time, transport, etc.) cost to conduct a VL test is ~ $35·38 site MSF’s reagent costs in Malawi. We also estimated the commodity cost of a VL test to be ~ $11.50 USD as a minimum, provided by the Malawi Ministry of Health Laboratory Specialist.

We multiplied the estimated number of VL sample redraws against the estimated cost per test to determine the potential cost savings that VLRR would introduce by returning the results within the clinically relevant period for the 11 EMR-enabled sites. We then performed this same analysis and predicted the potential cost savings if VLRR were to be scaled at the national level. The lower and upper bound cost savings formulas are show below:

- Lower Bound: (total # VL tests per year) x (% of tests redrawn due to processing delays) x (minimum commodity cost of VL test)
- Upper Bound: (total # VL tests per year) x (% of tests redrawn due to processing delays) x (comprehensive cost of VL test)

## Results

We have categorized our findings based on TAT, open rates, and feedback from clients and HCPs from the mixed methods study. Often sex and age are not captured in the LIMS system; however, for clients with age and sex reported, there were more female (29%) than male (19%) VLRR clients and most VLRR clients were aged 35–44 (15%). Age was confirmed with clients by HCPs at time of consent to ensure eligibility, however this information was not systematically recorded at that time, as we anticipated LIMS data to be more complete. Additional details on sex and age can be found in Table 2 and site-specific details can be found in Table 3.

**Table 2:** VLRR client demographics.

Sex	Result
• Female	1177 (29%)
• Male	776 (19%)
• Not reported	2114 (52%)
**Age (years)**	
• 18-24	57 (1%)
• 25-34	268 (7%)
• 35-44	603 (15%)
• 45-54	592 (15%)
• 55+	370 (9%)
• Null/data entry error	2177 (52%)

### 1) Turnaround time

Over the life of the intervention, HCPs successfully registered 4,067 clients (Table 3). Of those registered clients, 3584 (88%) received an SMS (Table 3). Some clients did not receive an SMS due to initial data entry error of client details at initial registration. The average TAT in Malawi for the status-quo process using EMR data from 249 sites was 85·9 days (from sample collection to the client’s next return visit).

**Fig 2:**
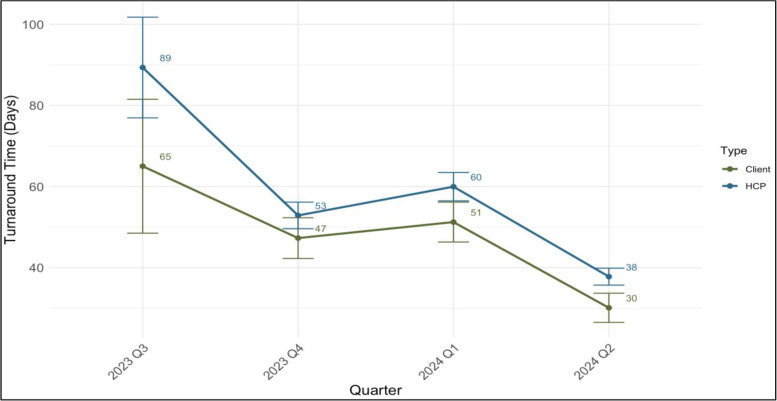
Average turnaround time by quarter and user type with 95% CI.

For VLRR, the average TAT for results return (sample collection to results receipt) at all 14 sites improved over time for both clients and HCPs. In the last quarter of 2023, the average TAT was 47 days for clients and 53 days for HCPs. By the end of Q2 2024, the average TAT reduced to 30 days for clients and 38 days for HCPs (Fig 2). The overall average TAT from Q3 2023 to Q2 2024 was 49 days for clients and 56 days for HCPs. We report from Q3 2023 to Q2 2024 because in Q2 2023, we resolved a back-end synchronization issue delaying results return as well as implemented an improved user workflow to streamline the process of checking results for clients.

For the 11 VLRR sites with EMR, the client’s TAT (sample collection to next facility visit) pre-VLRR enrollment was 129 days from January 2019 to May 2024. After VLRR enrollment, these same clients’ TAT reduced to 48·5 days from Q3 2023 to Q2 2024 to view their results. This is a 62·4% improvement in TAT for those clients who enrolled in VLRR at EMR sites, as non-EMR-enabled sites were excluded from this analysis due to no pre-VLRR data being available.

### 2) Application open rates

**Fig 3:**
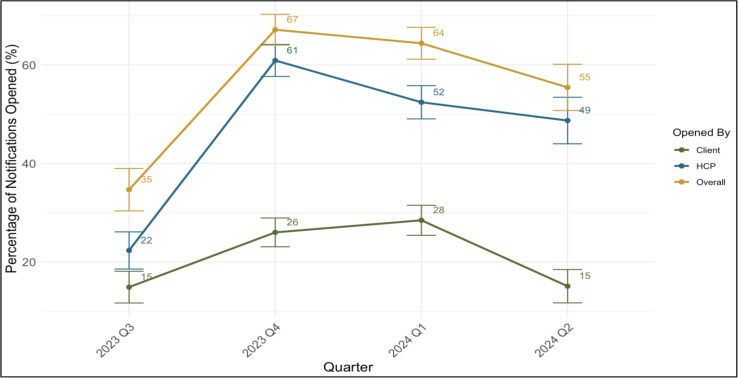
Quarterly open rates by user type with 95% CI.

The overall open rate, or the rate a result was opened by either a client or HCP, from July 1, 2023 to June 30, 2024 was 60%. Over this same period, the individual open rate for clients and HCPs was 25% and 50%, respectively (Table 3). For clients, quarterly open rates stayed relatively consistent from Q3 2023 to Q2 2024 with only slight increases in Q4 2023 and Q1 2024 (Fig 3).

However, HCPs had more variability in their open rate compared to clients over the same period with a big jump from Q3 2023 to Q4 2023 (Fig 3), which then slightly declined over time. Throughout the project, the study team along with DHA officials held quarterly site supervisions to engage with HCPs and identify if they were having any issues with the platform. The study team did not interact with clients directly throughout the life course of the intervention to protect client confidentiality.

### 3) Mixed methods evaluation

The findings from this evaluation support the quantitative findings of decreased TAT and improved open/access rate to results.

#### Client results.

We interviewed 117 clients across the 14 facilities. Of these, there were slightly more females than males (67 (57%) vs. 50 (43%)). The median age for clients interviewed was 44 years, with a range of 18–74 years. The median length of time a client was on ART was ~ 12 years, and the median length of VLRR enrollment was 4 months (Table 4). All clients, except one, indicated they had access to a phone. When asked how long clients typically waited for their paper VL results, the average wait time was 15 weeks across facilities, but some waited up to 48 weeks to receive their paper results.

### Theme 1: Favorability for VLRR and would they recommend it to others

Overall, clients expressed favorable satisfaction for the platform and would recommend it to others who need to receive a VL result. Clients noted that the application especially helped them to receive their results faster and to save time and money avoiding transport and travel to and from the facility (quote 1). Clients also expressed liking the ability to access their results from anywhere, especially at home. This meant they could avoid missing work for an additional/long visit to the clinic to solely receive their results. Clients also found the application motivating because it provided them confirmation that taking their medication was working to suppress their disease (quote 2). Further, most clients felt they received enough support from HCPs to access and use the platform adequately.

*Quote 1: “It’s better because you can receive results while you are at home through your phone even the results are still at the lab. We used to walk long distance coming to the hospital only to have access to results, for instance 10 kilometers to and from.” – Client, Mzimba District*

*Quote 2: “It is encouraging for me to continue taking medicine after seeing my results.” – Client, Machinga District*

### Theme 2: VLRR ease of use and perceptions of the platform

When asked if they thought VLRR was easy to use, clients overwhelmingly answered, “yes”. Eighty-nine percent of clients said the VLRR registration process was easy. Once clients were registered and started receiving results, 91% of clients said it was easy to access their results through VLRR. One client said, “its easy to know your results (client, Rumphi District Hospital).” A few clients struggled to remember their PIN or struggled with literacy and digital literacy, in general. For those with rudimentary digital literacy, they found it easy to access their results on the platform. Clients expressed surprise that they could receive their results on time/quickly and felt it gave them the ability to take proper action based on their results. Other clients expressed a sense of agency in their care, getting to know their results on their own and being able to go to their provider already knowing their results (quote3).

*Quote 3: “We know the results early before the 6 months so we can take action either visiting the facility if result is high or being stress free if the result is fine.” Client, Lilongwe District (Urban)*

### Theme 3: VLRR and privacy

We also sought to understand clients’ perceptions of VLRR and privacy. While most clients had no concerns about privacy (quote 4) and indeed expressed that VLRR increased their privacy “the platform provides privacy to us” (client, Lobi Health Centre), while a small number of clients expressed a level of concern surrounding their privacy and VLRR, especially if someone used their phone. However, many caveated this concern, by noting that the PIN eased their concerns since no one else knew their PIN and could access their results (quote 5).

*Quote 4: “I am confident while receiving the results on my phone because I alone get to see the results and it’s me and am the one to interpret the results that has to see the SMS.” Client, NkhataBay District*

*Quote 5: “At first my concern was that if I receive a message and someone went to use my phone she or he can see the message, but due to pin, he or she cannot have access to the message.” Client, Lilongwe District (Urban)*

### Theme 4: Client feedback on the platform

When asked whether they had feedback about VLRR, clients overwhelmingly liked and wanted to keep using the platform and liked how easy it was to access their results on their phones. Additionally, they wanted their friends and family to have access to it. Clients preferred VLRR to paper results because it saved them time by eliminating the need to travel to the clinic, saved them money by avoiding missed work, and provided them with fast results. They also got excited about knowing their results before visiting the clinic and expressed it gave them more agency to act if a clinical change was necessary. A few clients recommended simplifying the system, especially if they forget their PIN. Other clients suggested including additional reminders on the platform when their result was ready.

*Quote 6: “People who have phones should join this platform because they will receive their results earlier rather wasting time travelling to access the results.” – Client, Mulanje District*

### HCP results

We conducted interviews with 67 HCPs across the 14 facilities. The sample included 35 (52%) males and 32 (48%) females. All HCPs in our sample were a part of the VLRR training and had registered clients.

The median age of HCPs interviewed was 39 years, with a range of 23–59 years. The median amount of time HCPs had worked in HIV care was about 9 years and the average length of time using VLRR was 4 months (Table 4). The most common cadres represented were nurses (29%) and health diagnostic assistants/treatment supporters (32%). Table 5 presents a comprehensive list of the health cadres represented in the sample. About 50% of HCPs had some level of college education.

**Table 5:** List of cadres using VLRR and interviewed in the mixed methods study.

Cadre	N (%)
Health Diagnostic Assistant/Treatment Supporter	21 (32%)
Nurse (ART, Midwife)	19 (29%)
Clerk (ART, Data, ICT)	8 (12%)
Clinical Officer	5 (8%)
Assistant (Linkage, Medical, Midwife)	3 (5%)
Expert Client/Volunteer	3 (5%)
Attendant (Hospital, Patient)	2 (3%)
Supervisor (HTSS, Lab)	2 (3%)
ART Clinician	1 (2%)
Councilor (HTS)	1 (2%)
Facility In-charge	1 (2%)
Not reported	1 (2%)
**Total**	**67 (100%)**

### Theme 1: VLRR supported client care

Overall, HCPs supported the VLRR application and 97% said they were likely/very likely to recommend the application to other HCPs. Eighty-eight percent of clients thought results were easy to access through VLRR and 97% of HCPs thought the VLRR application improved client access to results (quote 7).

*Quote 7: Clients get results early while at home. They don’t come to the hospital frequently as we supply them with 6 months ARVs. HCP, Zomba District*

When reflecting on what made VLRR successful, HCPs stated that the VLRR application supported clients to receive their results quickly and easily and allowed them to access results anywhere, especially at home. HCPs also said it supported clients receiving results because they had to do less tracing since clients came back earlier than their next scheduled appointment, if unsuppressed (quote 8).

*Quote 8: “Now clients come on their own after seeing their results at home, in the past we had to go look for them because they did not have access to results at home.” HCP, Mulanje District*

### Theme 2: Challenges addressed by VLRR

Notable challenges of the paper-based system for healthcare providers included long TAT, long clinic visits/travel time, and lost/missing paper results (quote 9). When asked if they felt the VLRR platform addressed the challenges of the paper-based process nearly all said yes. Some of the challenges of the paper-based system addressed by VLRR included reduction of TAT, reduction of client travel time to and from the clinic, and that increased patient action and knowledge of results before their next scheduled appointment. The platform also addressed a common issue of missing results, since results cannot be lost digitally and can be accessed at any time (quote 10). Additionally, most HCPs strongly agreed that it was easy to enter client information and found results easy to access through VLRR.

*Quote 9: “Results come early than before, clients access results in good time and also it reduced our workload.” HCP, Dedza District*

*Quote 10: “Because clients are getting their results in good time and we are able to follow up with clients whose VL is high before their next schedule for hospital visit. We manage them in good time. Missing results challenge has been addressed as well.” HCP, Zomba District*

### Theme 3: HCP workload

HCPs said VLRR reduced/simplified their workload by reducing the number of clients coming to the facility to get their results and said it enhanced their work quality. Many HCPs noted their favorability for the platform because clients knew their results, which helped them to build trust with their clients by enhancing the reliability of clients receiving their results on time.

*Quote 11: “It has simplified our work since the client is able to view their results and know what to do (coming for help here when viral load is high).” HCP, Mulanje District*

*Quote 12: “It has highly improved our work, we are no longer panicking with long queues of clients.” HCP, Lilongwe District (Rural)*

### Theme 4: VLRR Continuity, Scalability, and Feedback

Additionally, nearly all clients and all HCPs said the platform should continue (quote 13) and should be scaled to additional facilities and communities (quote 14). We asked both clients and HCPs why or why not they would want VLRR to continue and both said it reduced TAT for results, both said it was a good initiative, and the technology was useful, HCPs said it reduced their workload, and clients liked the time and money it saved them. Further, both HCPs and clients noted that with the paper-based process, clients often would come back to the facility and their results would not be ready. This reduced client trust in the facility as well as reduced their desire to redraw their test and ensure they were truly virally suppressed, thereby affecting clinical care and outcomes. Notable feedback from both clients and HCPs was a request to reduce the amount of time it took to register a client in VLRR. Clients said it felt long, and HCPs said this process sometimes added to their workload. Despite this, resoundingly, HCPs and clients wanted others to access this platform and wanted it to be scaled nationally.

*Quote 13: “Just want to encourage you to continue [the platform], this program is helping me and my friends.” Client, NkhataBay District*

*Quote 14: We should… “train more HCPs and scale up this program in many facilities.” HCP, Machinga District*

#### 4) Cost analysis - redraws.

The VLRR intervention also has a future opportunity to provide system wide benefits and cost savings by reducing the overall laboratory backlog through the reduction of unnecessary redraw tests due to long TAT. According to EMR data, in Malawi, there are approximately 50,340 VL tests drawn each year at the 11 EMR-enabled VLRR sites. Approximately 30% (15,100) of those tests were avoidable redraws taken due to long TATs, which led to clinically irrelevant VL results. This 30% accounts for virally suppressed clients who had two tests within a 5-month period. We selected this 5-month cut off as DHA guidelines indicate suppressed clients are only required to be tested at intervals ranging from every 6 months to every 4 years, depending on client clinical history. If this 30% was avoided through expedited turnaround time of results, this would result in a potential cost savings of $173,650 to $534,238.

At the national level, Malawi draws about 675,000 VL tests per year. Of those 675,000 approximately 25–28% of those are avoidable redraws. Using the MSF comprehensive cost of $35·38, savings would be $6·7 million. Using the reagent cost of $11·50 provided by DHA officials, the minimal cost savings would be $1·8 million.

## Discussion

Adequate clinical decision-making relies on the timely return of laboratory results [1–3]. Delayed results inhibit clinical care, increase the cost and burden to HCPs and the healthcare system, and deteriorate the trust between provider and client [5]. WHO emphasizes the importance of VL monitoring for improving clinical outcomes [7]; however, adequate VL monitoring is not feasible if results are lost, delayed, or required to be redrawn. The VLRR intervention is a simple, but efficient digital health intervention that leverages existing government structures and existing back-end architecture in Malawi to improve TATs of laboratory results and increase access to results for both clients and HCPs faster than the paper-based process.

Prior to VLRR, Malawi had increased access to annual HIV VL testing to improve the quality of care for clients [9]. However, the VL results were still returned to clients in person, leading to delays, lost results, and missed opportunities for timely clinical interventions. This had significant implications for unsuppressed clients requiring adherence counseling or regimen changes and created additional backlogs at laboratories. VLRR addressed these issues by significantly reducing the turnaround times for VL results and ensuring both HCPs and clients could access their results more quickly and reliably from any location.

In addition to decreasing TAT, this intervention also intended to create a user-friendly digital solution that would increase client and HCP access to results, which we assessed through open rates. We consider the overall open rate to be more clinically relevant as action will be taken by *either* the client or HCP if needed. For example, if a client opens their results and finds they are unsuppressed, they are guided to visit the clinic as soon as possible. Similarly, if a HCP opens a client’s result and finds a client to be unsuppressed, they will send an expert client or community health worker to bring the client back to the facility to receive enhanced adherence counseling or be switched to 2nd line therapy, as appropriate.

To our knowledge, VLRR is the first application to leverage a combined SMS/USSD results return platform to clients in low- and middle-income countries. Because VLRR is the first intervention of its kind, there are no directly comparable open rates to measure success. However, we sought to understand open rates of digital applications providing test results to clients in both low and high-income countries [14,18–27] and assess how VLRR compared to these. The open rates for high-income countries ranged from 21% to 57%. The VLRR overall open rate was about 15% to 51% higher, compared to the best [25] and least [23] performing applications in these countries, respectively. For low-income countries, most interventions did not report an open rate, except for one smartphone application, which had a high open-rate rate of 78% [25]. While not directly comparable, VLRR’s overall open rate (60%) performs better or on par with reported smartphone and SMS interventions.

The faster TAT and usage rate of the VLRR platform are supported by the qualitative findings, which demonstrate the overall satisfaction that clients and healthcare providers have with the VLRR platform. Clients and healthcare providers alike appreciate how fast results are returned to them and the ease with which they can access them. One of the main objectives of VLRR was to enhance the speed at which clients and HCPs accessed VL results. The mixed-methods evaluation confirms that both clients and HCPs noticed a difference in TAT, liked the ease with which they could access results, and the benefits of accessing results at any time from any location, in private.

Further, the VLRR digital solution demonstrates the potential for system-wide benefits and essential cost savings by offsetting the overall laboratory backlog by reducing unnecessary redraw tests necessitated by long TATs. Nationally, this equates to $1·8-$6·7 million in potential cost savings. National scale of the platform and further cost analyses will be critical in the future to estimate true cost savings from digital results return. These cost savings have implications for the government, districts, facilities, and ultimately, clients.

The VLRR platform was designed with broadly accessible technology and adaptable architecture, allowing for seamless expansion to accommodate other functions. The VLRR platform can be leveraged for additional use cases including other tests (e.g., TB, malaria, HPV), appointment reminders, precision nudging to recheck results, and an AI chatbot for smartphone users. These use cases could be added to the platform with minor tweaks to the backend architecture and provide opportunities to lower average care costs by improving efficiency and reducing the excess burden of disease through enhanced clinical continuity. The platform extensions could leverage the existing VLRR frontend and backend architecture. These additional use cases could increase access to laboratory results and improve the standard of care for clients using this simple technology.

The success of the VLRR platform in both EMR- and non-EMR-enabled facilities demonstrates its feasibility in diverse clinical settings, including resource-limited and rural environments where infrastructure is often minimal. This makes VLRR particularly well-suited for national scale-up efforts in Malawi and other low- and middle-income countries facing similar challenges. The integration of VLRR into national HIV programming could strengthen the effectiveness of viral load monitoring systems by reducing turnaround times, minimizing unnecessary redraws, and improving client follow-up. As a scalable and low-cost digital innovation, VLRR presents a compelling case for policy adoption and long-term integration into routine HIV care delivery.

At national scale in Malawi, integration would involve maintaining the existing API connection between the LIMS database and the VLRR platform, continuing the partnership with the two primary mobile network operators, and leveraging the Ministry of Health’s dedicated shortcode (*929#) to ensure broad accessibility across both smartphone and non-smartphone users. In other countries, a similar approach could be replicated by linking the platform to national laboratory databases, securing a government-owned shortcode, and partnering with major telecom providers, with minimal modifications to the backend to account for country-specific systems and workflows.

### Limitations and lessons learned

The VLRR TAT was variable to many exogenous factors that could not be controlled by the study team. Some of these factors included telecommunication company service disruptions, nationwide power outages, client literacy rates, clients changing SIM cards, commodity stockouts, and challenges with the LIMS server. During periods of downtime, we would notify all HCPs, via WhatsApp, that VLRR was down and would be back up as soon as possible and communicated as best we could on how the issue would be resolved.

To address challenges around literacy, we tried to simplify the process by implementing the PIN workflow for clients. This would reduce the amount of information they would have to enter to access their results, but still respect their privacy and maintain confidentiality because the PIN they chose was specific to them.

We also experienced data quality challenges with EMR data and incorrect data entry of ART numbers and sample barcodes. We matched as many samples as possible based on multiple client identifiers. However, this, at times, created challenges to calculate the most accurate TAT. Additionally, non-EMR sites do not have a baseline TAT besides past studies that have estimated TAT. So, it becomes a challenge to compare VLRR’s TAT to an existing metric for Malawi. However, we utilized the data provided by the specimen transport service to estimate the TAT for non-EMR sites to have a rough estimate for comparison.

Another key limitation of this evaluation is the high proportion (52%) of missing demographic data, particularly sex and age, in the LIMS data. While these variables were confirmed during client enrollment, they were not systematically documented outside of the LIMS, limiting our ability to analyze trends by demographic subgroup. This missingness could bias our findings if demographic characteristics are associated with differences in TAT, open rates, or platform use. Future evaluations should ensure demographic data are captured at enrollment and cross-verified with LIMS to enable robust subgroup analyses.

Lastly, it is necessary to note the assumption made when estimating cost savings from unnecessary redraws. This cost savings estimate assumes clients return to the clinic based on the national testing algorithm indicating when a client should return for their next VL test. We acknowledge the potential for misclassification of a client that requires a redraw due to clinical irrelevance due to this assumption.

## Conclusion

Despite the challenges faced during the VLRR intervention, the application has achieved many more successes for both HCPs and clients accessing VL results faster than the status quo paper-based processes. The VLRR application demonstrates that long TATs can be mitigated with simple digital solutions. VLRR has demonstrated the improvements that can be made by targeting gaps in the process of sample collection to results return. To further enhance uptake and national scale of the platform, VLRR can be integrated into existing point-of-care systems within Malawi (e.g., the EMR) to continue to reduce TAT and support providers to treat clients adequately and timely, to continue improving clinical outcomes long-term. Integration into the EMR would also reduce the time at registration for clients and HCPs as all client information is already captured in the EMR. Further, this technology can be easily adapted and leveraged in other countries to support faster results return for HIV-positive clients to support the improvement of global suppression rates.

## Supporting information

S1 File Visual abstract. (DOCX)

S1 Data VLRR Data. (CSV)
