# Supplementary figures and images for: Mobile phone infrastructure provides evidence of improved HIV viral load monitoring in Malawi
Source: PLOS Digit Health. 2026 Jan 21;5(1):e0001094. doi: 10.1371/journal.pdig.0001094 (PMC12822985; doi:10.1371/journal.pdig.0001094)

**Visual Abstract:**


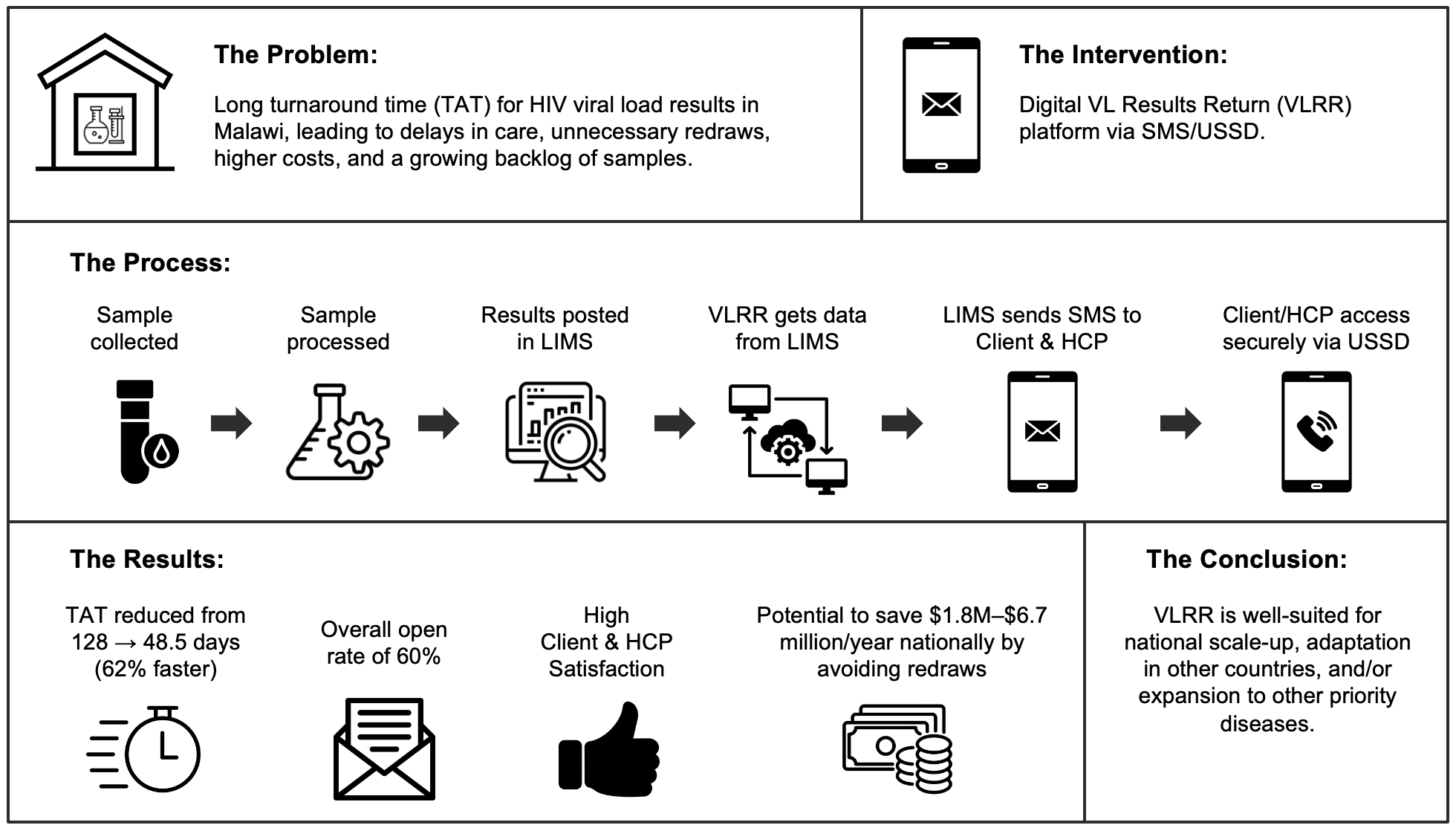

Supplement: S1 File — (DOCX) [file pdig.0001094.s001.docx]
